# Five keypoints in Foucher flap planning and harvesting: Retrospective case series study and literature review^1^

**DOI:** 10.1016/j.jpra.2025.08.031

**Published:** 2025-09-02

**Authors:** Mariagiovanna Lombardi, Martina Ponzo, Vincenzo Iazzetta, Ludovica De Gregorio, Giorgio Squarcia Neri, Annachiara Cavaliere, Fabrizio Schonauer

**Affiliations:** a Department of Plastic and Reconstructive Surgery, Università degli Studi di Napoli Federico II, Via Pansini 580131, Napoli, Campania, Italy; b Department of Plastic, Reconstructive and Aesthetic Surgery, “Campus Bio-Medico” University of Rome, Rome, Lazio, Italy

**Keywords:** First dorsal metacarpal artery island flap, Foucher flap, Kite flap, Thumb reconstruction

## Abstract

**Introduction:**

The Foucher flap, based on the first dorsal metacarpal artery, is an effective solution for thumb reconstruction following trauma or oncologic resections. This technique allows for the preservation of thumb length and volume while keeping sensitivity and functionality. The success of this surgical approach depends on meticulous planning and execution. This retrospective study analyzes five key aspects related to flap planning and harvesting: defect location, preoperative vascular pedicle identification, flap design, surgical dissection and pedicle insetting.

**Patients and Methods:**

A total of 24 patients who underwent Foucher flap thumb reconstruction between 2013 and 2023 were evaluated. The artery was preoperatively identified using Doppler imaging in all cases. Flap transfer was performed either through subcutaneous tunneling or skin incision, depending on the pedicle's characteristics. Functional and aesthetic outcomes were assessed through the Kapandji score, two-point discrimination and patient satisfaction surveys.

**Results:**

At an average follow-up of six months, all flaps survived entirely. The mean Kapandji score was 8.17 and two-point discrimination in the skin paddle averaged 5.92 mm. Patient satisfaction was high, with 79 % rating their outcome as ``excellent'' and 21 % as ``satisfactory.'' Clinical evaluations confirmed favorable functional and aesthetic results.

**Conclusions:**

The literature review and clinical data support the Foucher flap as a reliable technique for thumb reconstruction. Careful preoperative planning and precise execution are essential to optimizing functional and aesthetic outcomes, minimizing complications and improving patients' quality of life.

## Introduction

The loss or damage to the thumb represents a significant challenge for reconstructive surgery, given the importance of this finger in hand functionality and in performing daily activities. The thumb, in fact, is responsible for about 40 % of the hand's functions, critically contributing to the grip and manipulation of objects. The surgical techniques to repair thumb injuries depend on the type of injury, its location and severity. This ranges from superficial injuries that can be treated with grafts, etc., to deeper injuries that can be treated with homodigital flaps to more complex ones for which heterodigital and distant flaps can be used, up to the indication for free flaps.

Among the various techniques available for thumb reconstruction, Foucher flap has stood out as a particularly effective option. This approach uses a pedicled neurovascular flap, taken from the dorsal region of the index finger and based on the first dorsal metacarpal artery (FDMCA), to preserve not only the length and bulk of the thumb but also to preserve its sensitivity and functionality. Introduced in 1979 by G. Foucher,^1^ this technique has revolutionized the field of hand surgery, offering patients significantly improved functional and aesthetic results.^1^, ^2^, ^3^

So, this article does not propose a “new” flap, but rather an evolution of the Foucher flap: it expands its indications and dimensions, systematizes technical steps that were previously scattered across individual studies and, most importantly, provides an operational framework based on a dedicated case series and recent literature, including a systematic review of 12 studies (2014–2024). This allows for a direct comparison between the authors’ results and those reported in the literature and helps derive practical guidelines. In 18 out of 24 cases, the classic flap was extended with a triangular skin expansion at its base, which facilitates insetting and reduces tension on the pedicle—a technical detail not previously described. The article distills clinical experience into five key steps—defect location, preoperative arterial identification, flap design, dissection and rotation arc/insetting—proposing a true decision-making algorithm for the Foucher flap.

### Anatomy

The vascular axis of the flap is represented by the first dorsal metacarpal artery (FDMCA), accompanying veins and a cutaneous sensitive branch of the radial nerve. The first dorsal metacarpal artery has a diameter of 1.0–1.5 mm and can have either a superficial (fascial) or deep (muscular) course.^4^ The superficial course originates from the radial artery (77%) at the level of the carpi radialis longus extensor tendon , before entering the first dorsal interosseous muscle or from the ulnodorsal digital artery of the thumb (13%). In 33 % of cases, it communicates with the second palmar metacarpal artery at the neck of the second metacarpal bone. The deep variant arises from the radial artery and passes between the two heads of the first dorsal interosseous muscle. In some cases, both vessels (the superficial and muscular branches) are present.^4^

The FDMCA vascularizes the proximal phalanx and the MCP joint of the index finger, the first web space and the dorsal surface of the thumb Table 1.

**Table 1:** General characteristics and outcomes of patients.

Pt.	Sex/age (year)	Etiology	Type of thumb defect	Follow-up (month)
1	M/44	Trauma	Dorsal side DP+PP	6
2	F/32	Trauma	Dorsal side DP	9
3	F/41	Post-oncological	Cuff pattern on DP	7
4	M/38	Trauma	Dorsal side DP+PP	7
5	M/42	Trauma	Amputation of DP	5
6	M/33	Trauma	Dorsal side PP	5
7	M/34	Trauma	Dorsal side DP	4
8	F/39	Post-oncological	Amputation of DP	8
9	M/35	Trauma	Volar side DP	7
10	M/37	Trauma	Volar side + tip DP	7
11	M/36	Trauma	Dorsomedial side DP+PP	4
12	M/40	Trauma	Medial volar side DP+PP	9
13	M/41	Trauma	Dorsal side DP+PP	8
14	F/36	Trauma	Dorsal side DP	5
15	F/44	Post-oncological	Cuff pattern on DP	5
16	M/38	Trauma	Dorsal side DP+PP	5
17	M/42	Trauma	Amputation of DP	5
18	M/35	Trauma	Dorsal side PP	6
19	M/39	Trauma	Dorsal side DP	6
20	F/33	Post-oncological	Amputation of DP	5
21	M/37	Trauma	Volar side DP	7
22	M/40	Trauma	Volar side + tip DP	4
23	M/34	Trauma	Dorsomedial side DP+PP	5
24	M/43	Trauma	Medial volar side DP+PP	6

Abbreviations: DP, Distal Phalanx; F, Female; M, Male; PP, Proximal Phalanx; Pt. Patient number;.

## Materials and methods

We have collected the procedures carried out from 2013 to 2023, concerning patients treated for injuries to the thumb, looking for “Keypoints” in Foucher flap planning and harvesting. Out of 45 flaps, 24 patients were treated with the Foucher flap.

Diabetes and smoking increase the risk of flap complications but are not absolute contraindications. They require careful vascular assessment and preoperative optimization. Diabetes affects microcirculation and healing, while smoking reduces blood flow through vasoconstriction and endothelial damage. Peripheral vascular disease and vessel injuries may be contraindications and active infections or skin conditions at the donor site are absolute contraindications.

The average age was 37 years, predominantly male with a traumatic etiology. 12 of the cases showed trauma to the dorsal region, four patients with distal phalanx amputation (IPD joint preserved), six patients with injury to the palmar region and two patients with distal phalanx injury.

A visual analogue scale (VAS)^5^ was submitted to patients to evaluate their satisfaction with the appearance of their thumb after surgery. The result was rated as ‘‘not improved,’’‘‘improved,’’‘‘satisfactory’’ and ‘‘excellent.’’

Cosmetic results were also evaluated by seven different observers (five plastic surgeons and two nurses blinded with surgical details) with frontal, lateral, 3/4 left and 3/4 right views of photographs at 1 year follow-up. The general appearance of the thumb, its skin colour, its contour were considered. Observers reviewed the pictures and scored the results on a 5-point Likert scale^6^ that ranged from ‘poor result’ to ‘‘excellent result.’’ A mean score greater than 4 was considered as satisfactory result. The thumb opposition ability was assessed using the Kapandji score.^7^

The results were compared with those in the available literature. The review was performed by searching the PubMed, Medline and Embase databases as of April 24, 2024. We included only articles published within the previous 10 years (2014-2024).

A total of 117 records were screened and assessed for eligibility. Exclusion criteria encompassed case report, studies that had a caseload of <5 patients. The relevance of each article was determined by evaluating its title, abstract and full text. The search terms used were: First Dorsal Metacarpal Artery Island Flap, Foucher flap, Kite flap. 12 articles met the criteria for inclusion (Table 2).

**Table 2:** STUDIES INCLUDED IN THE REVIEW.

Authors	Title	Journal	Year of Publication
Zyluk A. et al^26^	Outcomes of Coverage of Soft Tissue Defects in the Thumb with a ``Kite Flap''	*Handchir Mikrochir Plast Chir*	2023
Günay AE et al^14^	Mid-Term Results of the First Dorsal Metacarpal Artery Flap for Thumb Defects	*J Hand Surg Asian*	2022
Moog P et al^24^	Donor Defect Morbidity of Intrinsic Flaps in the Posttraumatized Hand	*Ann Plast Surg*	2021
Al Lahham S et al.^25^	A Modification to Enhance the Survival of the Island FDMA Flap by Adding a Skin Bridge	*Plast Reconstr Surg*	2021
Chi Z et al.^23^	Routine closure of the donor site with a second dorsal metacarpal artery flap to avoid the use of a skin graft after harvest of a first dorsal metacarpal artery flap.	*J Plast Reconstr Aesthet Surg*	2018
Ghoraba SM et al.^13^	Outcome of Thumb Reconstruction Using the First Dorsal Metacarpal Artery Island Flap.	*World J Plast Surg*	2018
Kola N. et al^15^	Thumb Reconstruction Using Foucher's Flap	*J Med*	2016
Wang H. et al.^11^	Modified first dorsal metacarpal artery island flap for sensory reconstruction of thumb pulp defects	*J Hand Surg*	2016
Adani R. et al^16^	Reconstruction of Traumatic Dorsal Loss of the Thumb: Four Different Surgical Approaches	*Hand*	2019
Couceiro J. et al^19^	The First Dorsal Metacarpal Artery Flap Family: A Review	*Surg J*	2018
Shehata Ibrahim Ahmed M. et al^21^	Evaluation of versatility of use of island first dorsal metacarpal artery flap in reconstruction of dorsal hand defects.	*Asian J Surg*	2019
Küçükgüven A. et al^22^	Evaluation of versatility and outcomes of the first dorsal metacarpal artery flap in thumb defects.	*Ulus Travma Acil Cerrahi Derg*	2022
Webster N. et al^20^	Flaps Based on the Dorsal Metacarpal Artery.	*Hand Clin.*	2020

### Surgical procedure

After identifying the course of the FDMCA artery with the Doppler probe, debridement of the exposed area was performed with the help of surgical loops. The skin island was marked. Skin incisions were planned. During surgery, a tourniquet was inflated to 320 mmHg. The skin was elevated above the subcutaneous tissue of the pedicle. Sterile stitches were placed to hold the skin flaps. The skin island was then approached.. The flap was harvested above the peritenon layer.

The tourniquet was released and meticulous hemostasis was performed. At the thumb level, an extensor tendon graft was used when indicated (if required due to trauma). At the MCP joint level, a perforator extending down to the joint was gently dissected using bipolar forceps. The FDMCA was included in the dissection and lifted. The pedicle was usually left about 2 cm wide and include subcutaneous tissue, the dorsal metacarpal sensory nerve and two subcutaneous veins.

The flap was transposed to the defect through a subcutaneous tunnel or after opening a skin bridge. The flap was secured with 6/0 Ethilon sutures. The skin bridge opening was closed when indicated. The donor site was closed with a skin graft or a dermal substitute followed by skin grafting. When necessary, a Kirschner wire was used to stabilize the first IP joint. A protective palmar splint was applied to keep the hand and, most importantly, the thumb in a neutral position. Active mobilization began on the first postoperative day and continued for three months (Table 3).

**Table 3:** Results.

Pt.	Size of island flap (cm)	Insetting of the pedicle	Rotation angle (°)	
1	26,5	Skin incision	24	Tendon graft
2	13,2	SC Tunnel	25	/
3	19	Skin incision	25	Tendon graft
4	7,7	SC Tunnel	26	/
5	6	SC Tunnel	26	/
6	7,8	SC Tunnel	24	/
7	10	SC Tunnel	27	/
8	13,5	SC Tunnel	25	/
9	10	SC Tunnel	25	/
10	7,2	SC Tunnel	26	/
11	20	SC Tunnel	26	/
12	17,5	Skin incision	25	/
13	25,0	Skin incision	24	Tendon graft
14	12,5	SC Tunnel	25	/
15	18,0	Skin incision	25	Tendon graft
16	8,2	SC Tunnel	26	/
17	6,5	SC Tunnel	26	/
18	10,0	SC Tunnel	27	/
19	13,8	SC Tunnel	25	/
20	9,5	SC Tunnel	25	/
21	7,0	SC Tunnel	26	/
22	20,5	SC Tunnel	25	/
23	11,2	SC Tunnel	25	/
24	21,5	Skin incision	24	Tendon graft

Abbreviations: Pt. Patient number; SC, Subcutaneous.

## Results

**Figure 1:**
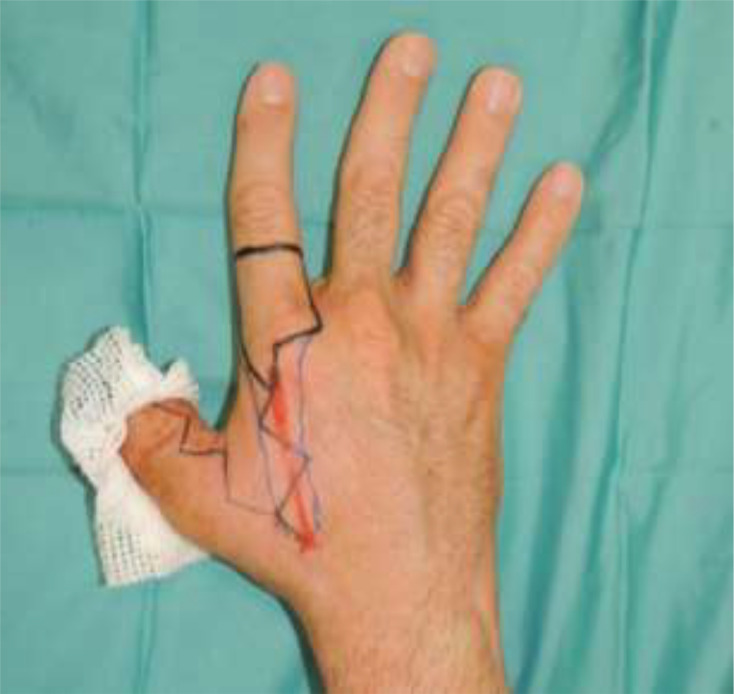
Foucher flap with Classic design island.

**Figure 2:**
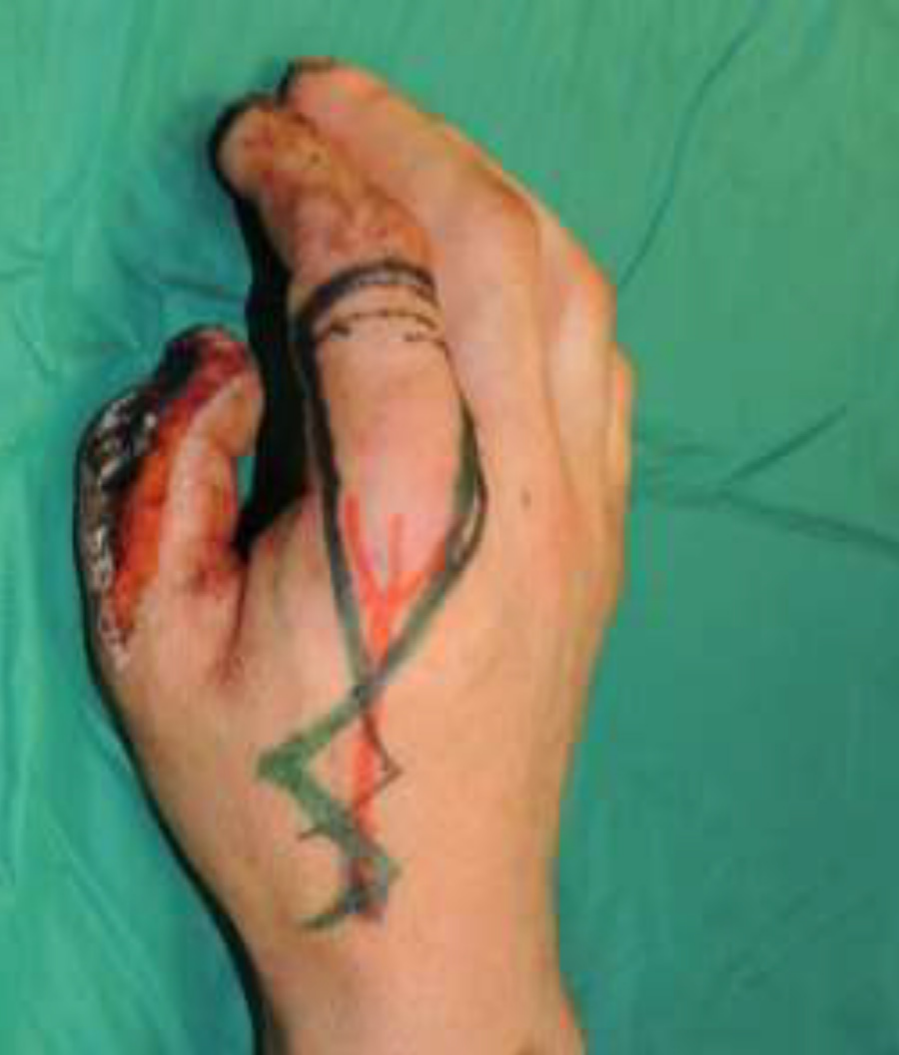
Foucher flap with Extended design island.

**Figure 3:**
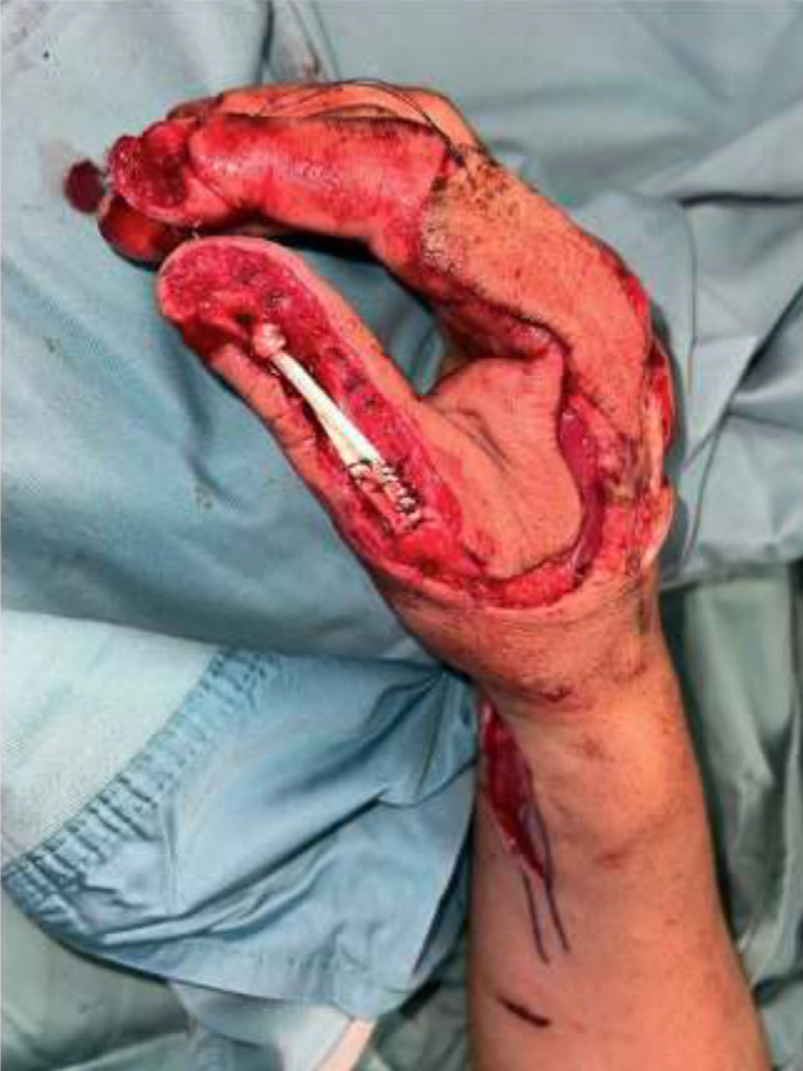
Tendon graft harvested from the palmaris longus tendon.

**Figure 4:**
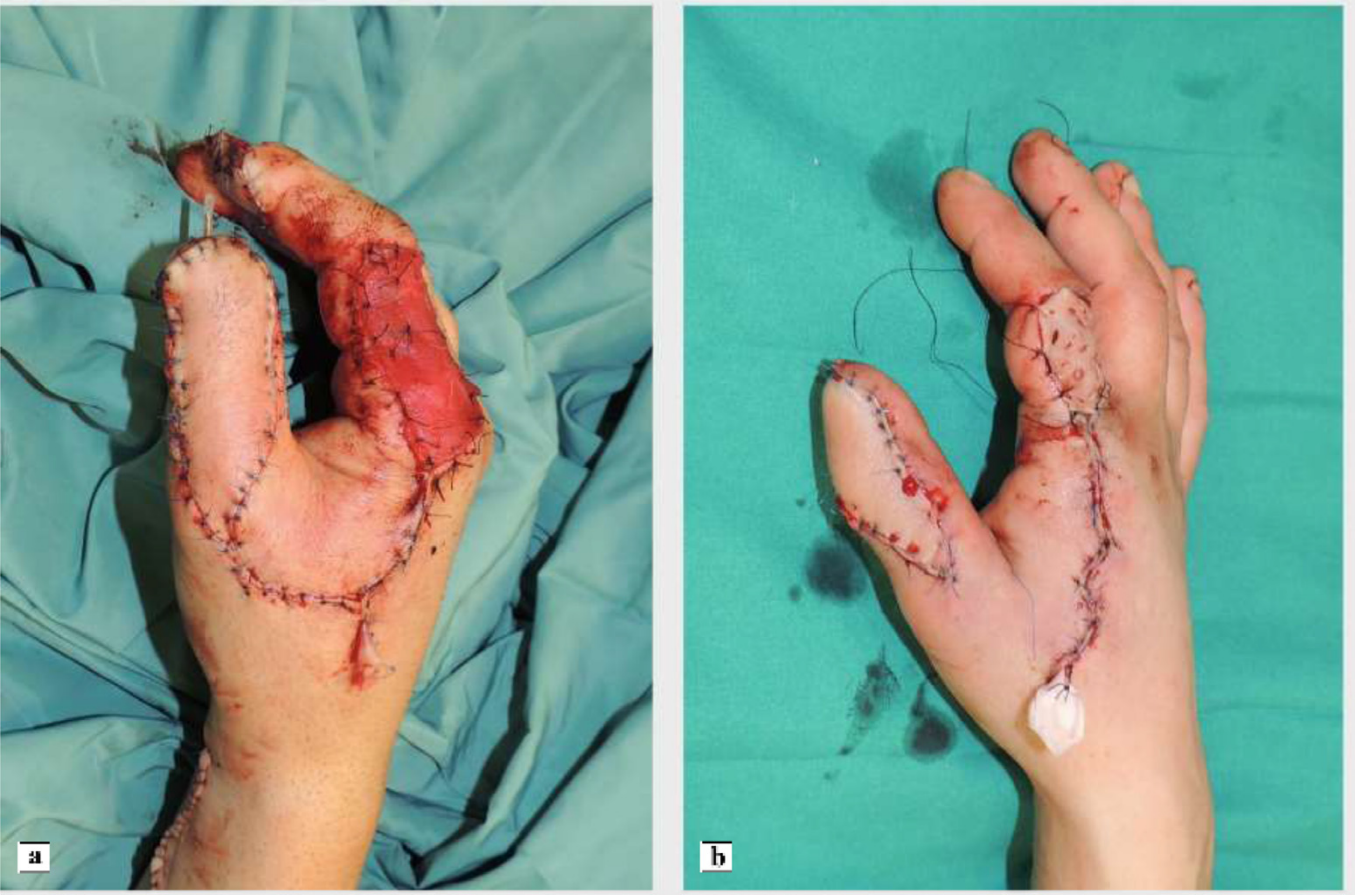
A) Pedicle coursethrough a skin incision. B) Pedicle was allocated in a subcutaneous tunnel.

**Figure 5:**
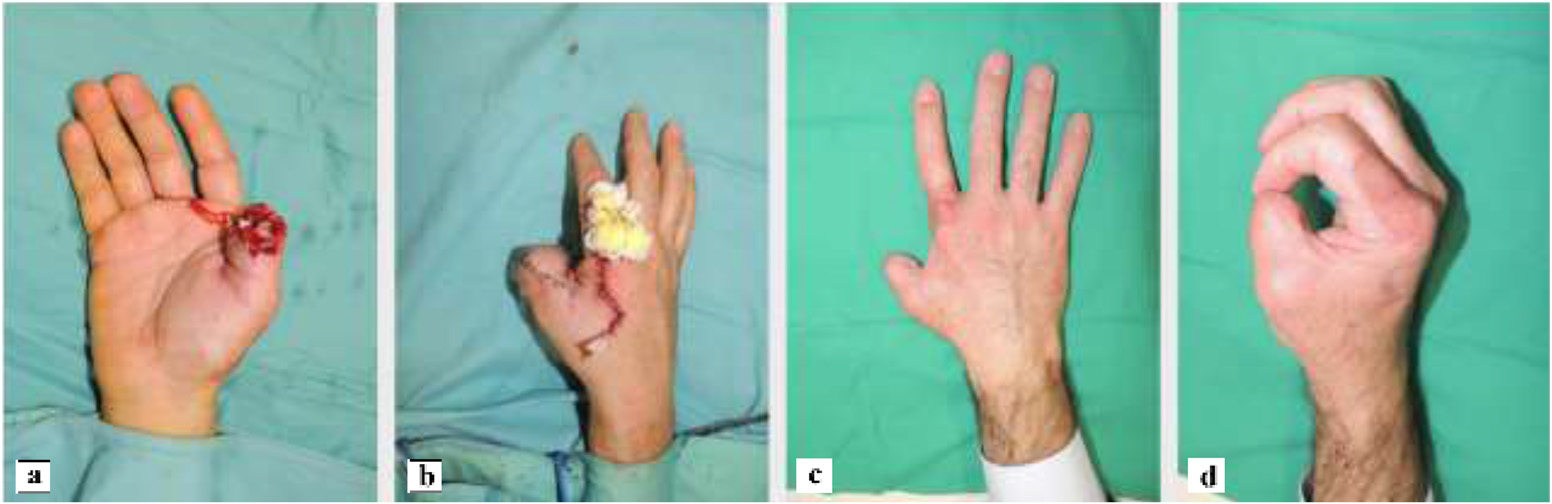
Case 1: a) amputation of the distal phalanx of the thumb. B) reconstruction with a Foucher flap with insetting through a subcutaneous tunnel and full-thickness graft on the donor site. C-d) 6-month follow-up with evaluation using the kapandji score.

**Figure 6:**
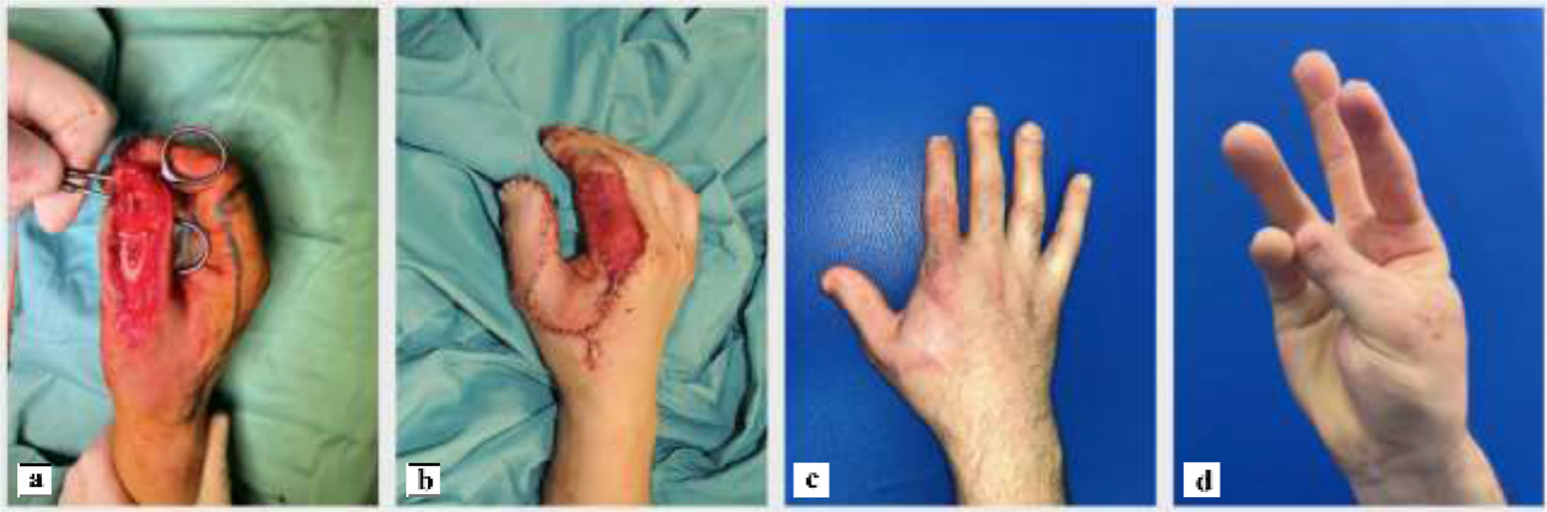
. case 2: a) Avulsion injury of the distal and proximal phalanx of the thumb b) reconstruction performed using an extended design Foucher flap with insetting through a skin incision; the donor site was reconstructed with a dermal matrix followed by a skin graft at a later stage. c-d) 3-year follow-up after surgery with evaluation using the Kapandji score.

The artery was preoperatively identified in all 24 patients, using Doppler examination. Out of a total of 24 patients treated, 18 required a flap with a classic design plus the addition of a cutaneous triangle at the proximal margin to improve insetting (Figure 1). Six patients needed an extended design flap (Figure 2). The mean flap area was 12.8 cm² [range 5.7 cm² - 24.5 cm²]. 5 cases required a tendon graft taken from the palmaris longus tendon (Figure 3). In 18 patients, the pedicle was moved through a subcutaneous tunnel, while in 6 patients a skin incision was performed so as not to overload the pedicle with too much pressure (Figure 4). The rotation angle of the pedicle averaged 25° [range 24°−27°]. A median follow-up of 6 months was conducted: all flaps survived entirely; the 2-point discrimination, in the middle of the skin paddle, was 5.92 mm (range, 2–10 mm); the mean Kapandji Score (Figure 5, Figure 6). was 8.17 [range 7–10] and mean VAS Score for postoperative appearance was "satisfactory".

Results were rated as ‘‘excellent’’ by 19 patients ( %), while the remaining 5 patients ( %) rated ‘‘satisfactory’’ their result.

Mean clinician’s panel evaluation was 4.3 for general appearance, 4 for skin colour, 4.5 for contour.

## Discussion

Reconstructive thumb surgery plays a pivotal role in the field of hand plastic surgery, addressing the complexities associated with the functional recovery of the hand following injuries or loss of the thumb. Analyzing data from scientific literature over the last 10 years, some key trends and considerations emerged regarding the application of this surgical technique for the reconstruction of hand injuries, particularly of the thumb.

Several flap options are available for thumb reconstruction depending on the defect’s location, size and depth. Moberg flaps are suitable for smaller defects, preserving sensation and function with minimal morbidity. The Quaba and modified Littler flaps are alternatives for specific regions but have limitations in sensitivity or aesthetics. Larger or complex defects may require microvascular free flaps, which involve higher morbidity and surgical complexity.

Thumb reconstruction using the dorsal arterial flap of the first metacarpal (FDMCA) is an area of growing interest in reconstructive surgery, thanks to studies by RJ Ratcliffe,^8^ Ahmet Ege,^9^ Ricardo Horta,^10^ which have provided significant results and innovations in the technique. RJ Ratcliffe^8^ conducted a study on 5 patients, with an average age of 55.4 years, who suffered from extensive injuries ranging from the tip of the thumb to the interphalangeal joint, demonstrating that the volar part of the thumb can be effectively reconstructed with this flap.^8^ The success in covering defects was 100 %, with patients reporting a gain in sensitivity from the dorsal surface of the index finger. A crucial point was early mobilization, which played a fundamental role in minimizing the loss of function.

In Ahmet Ege's work,^9^ 21 cases with defects varying from 12×18 mm to 20×40 mm, treated with flaps of length between 55–95 mm, were evaluated. Sensitivity outcomes, measured by the Semmes-Weinstein score, ranged from 3.61 to 4.31, indicating a good recovery of sensitivity in the flap. Wang H et al.^11^ used a flap innervated by the dorsal branch of the proper digital nerve and by the terminal branch of the superficial radial nerve. At the final follow-up, the average values of two-point static discrimination and the Semmes-Weinstein monofilament test in the study group were significantly different from those of the control group. Compared to alternative techniques, the FDMCa flap showed fewer disadvantages and better results in terms of sensitivity and functionality.

Hamdy A. et al.^12^ extended the discussion with a study on 5 patients, with an average age of 26.2 years, presenting lesions from 4 × 2.5 mm to 6.25 mm. Here too, flap survival was 100 %, with two-point discrimination tests showing sensitivity of 9–10 mm in the proximal part and 10–13 mm in the distal part of the thumb, underlining a good recovery of sensitivity. However, one case of postoperative venous congestion was observed, which was effectively managed.

From our experience, we have identified 5 key points to consider during the planning and execution phases to achieve the best possible outcomes: defect position and size, pedicle identification, flap design, surgical dissection, flap movement and insetting.

The first aspect to evaluate is the location and size of the defect. In our case series, 50 % of defects were located in the dorsal region of the thumb, 25 % in the volar region and 25 % involved amputation of the DP or cuffpattern. The dorsal region emerged as the most commonly affected area.^13^ The flap proved to be a valid alternative for reconstructing all the affected areas, extending even to the fingertip,^14^, ^15^, ^16^ and was also suitable for reconstructing tissue loss in the dorsal region of the hand.^21^

Another crucial step is identifying the **FDMA** (First Dorsal Metacarpal Artery), particularly its ulnar branch, which serves as the main pedicle of the flap and is located above the metacarpal bone of the index finger. As it passes the metacarpophalangeal joint, it divides into several small branches that supply the overlying skin, periosteum and tendons. The artery is identified by marking the first and second metacarpal bones and the FDMA is located between these lines with the assistance of a Doppler device.^11^^,^^17^^,^^18^

The FDMA is superficial, easily accessible and, in the analyzed series of 24 patients, was successfully identified in 100 % of cases using Doppler. This method, in fact, is not only fast and effective, but also radiation-free, does not require contrast agents, has minimal cost and can be repeated intraoperatively. In this context, angiography is not so much an alternative as a costly complement, indicated only when Doppler is insufficient, in complex cases or those with altered vascularization.

The absence of a preoperative Doppler signal is a contraindication for the use of these flaps.^19^ Additionally, the FDMA flap cannot be utilized if there is a radial artery injury in the anatomical snuffbox.^18^

This flap can also be designed on the dorsal aspect of the proximal phalanx of the thumb, utilizing the radial branch of the FDMA (FMDAr) for reconstruction of the first interdigital space.^18^

Once the arterial vessel is identified, the flap design can be outlined. It may follow a classic design, with the proximal limit at the level of the metacarpophalangeal joint, or an extended design^20^^,^In our experience, we added a triangular skin extension at the proximal margin of the flap to both designs. The flap is centered along the longitudinal axis of the ulnar branch of the FDMA (FDMAu), ensuring not to extend too far toward the volar side of the finger.

The dimensions of the flap should exceed the measured defect size by 15–20 % to account for post-harvest skin contraction.^18^, ^21^

The most commonly used incision for the cutaneous pedicle is the Lazy-S incision,^11^, ^19^, ^20^, ^22^ which reduces tension during closure and provides a good view during dissection. In our case series, we performed this incision in 14 patients, while in the remaining 10, a Zig-Zag incision^9^ was used.

Donor site morbidity is generally low, but not negligible. In our series, the donor site was reconstructed with a skin graft in 23 patients, while in 1 patient a dermal matrix was used, followed by a skin graft. There were no cases of chronic pain or significant functional deficits and the aesthetic outcomes were satisfactory. Literature shows higher morbidity when local flaps aren’t adequately mobilized. Potential complications include scar contracture, nerve injury and finger deviation if closure is not balanced.

An alternative approach was presented by Chi Z et al.,^23^ who employed a C-shaped incision at the pedicle to reconstruct the donor site with a reverse flap based on the SDMA, thus avoiding the need for a skin graft.^24^

The dissection phase is crucial for the success of the procedure. The vascularization of the FDMA flap is preserved when the entire interosseous muscular fascia is included, which eliminates the need for meticulous dissection of the artery, thereby reducing the risk of injury and flap loss.^25^ During dissection, it is important to consider that the FDMA may follow either a fascial or subfascial course.^15^, ^21^, ^26^

At the level of the second metacarpal, an important perforator (the distal communicating perforator between the dorsal and palmar metacarpal arteries) must be ligated. Special care is needed when elevating the ulnar portion of the flap, as excessive tissue excision in the interdigital space may cause contracture of the space.

Large subcutaneous veins encountered during dissection should be preserved and included in the flap to facilitate venous drainage. The flap can also be extended along the radial border of the third metacarpal to ensure additional venous drainage, since oneof the most common complications associated with this flap is venous insufficiency, which can lead to venous congestion, edema and related issues.

Al Lahham S et al.^25^ stated that adding a skin bridge to an island flap is more important for enhancing venous drainage than arterial input. In their study, they described a technique that addresses both issues by adding a 5 mm skin bridge that includes at least one vein to optimize drainage.

The insetting of the vascular pedicle represents the final step of the procedure and requires careful planning. Due to the limited length of this pedicled flap, accurate measurement of the rotation arc is critical. In our case series, the maximum rotation arc was 27° (mean 25,29°) and the anticipated site of insertion was carefully planned during flap design (Video 1). The pedicle can be placed through a subcutaneous tunnel or via a skin incision along the thumb.^20^, ^21^, ^26^

It is recommended to keep this subcutaneous tunnel wide to minimize potential compression effects on the vascular pedicle. The most common complication is necrosis of the distal portion of the flap, which may lead to further issues such as delayed wound healing, infection, or the need for additional surgical interventions.

If venous congestion occurs after performing an island flap, it may be possible to salvage the flap by widening or opening the tunnel towards the thumb.^19^

Foucher flap reconstruction has demonstrated excellent recovery of motor function and sensitivity in all studies. Sensory recovery is achieved through innervation provided by the superficial branch of the radial nerve (SBRN), which supplies sensation to the dorsal surface of the index finger and the second interdigital space. This nerve is harvested during the dissection of a sensory FDMA flap. However, its harvesting results in sensory deficits in the dorsal skin of the indexfinger.

At an average follow-up of 6 months, motor function recovery of the thumb was greater in patients who underwent early postoperative mobilization. Sensory outcomes for both the donor site and the flap were optimal in all treated patients.^24^

## Conclusion

In conclusion, the case analysis identified “five keypoints” for proper planning and execution of this surgical procedure:

• **Defect position**: This is a crucial factor for adequate planning. While the flap has proven effective in all areas of the thumb, its position influences the operational decisions in subsequent phases.
• **Preoperative artery identification**: Doppler examination is essential for identifying the artery and assessing the feasibility of the procedure. The presence of a Doppler signal is the determining factor for proceeding.
• **Flap design**: This can be performed in either the classic or extended form. Including the cutaneous triangle at the proximal margin and opting for S-shaped or zigzag incisions facilitate the proper insetting of the pedicle. The average maximum size of the flap reached in the analyzed studies was 15 cm^2^, while in our case, it was 24.5 cm^2^.
• **Dissection**: This is the most delicate phase of the procedure, requiring particular attention and precision to avoid complications. Dissection should be executed with the help of surgical loops.
• **Arc of rotation and pedicle insetting**: Studying these aspects is essential to minimize complications. The wide arc of rotation and the achievable size of the flap allow for the coverage of lesions in all areas of the thumb and even the dorsum of the hand.

These key points highlight the importance of a structured and precise approach to achieving optimal outcomes while ensuring the versatility of the flap in various clinical situations.

These studies emphasize the effectiveness of the FDMCa flap in thumb reconstruction and lead the way for important considerations regarding surgical technique, functional outcomes and the management of postoperative complications. The growing body of evidence supports a personalized surgical approach aimed at maximizing functional recovery and improving the quality of life for patients with significant thumb injuries.

## Funding

None.

## Ethical approval

Not required.

## Declaration of competing interest

None declared.

## Appendix Supplementary materials

Image, video 1 dummy alt text
